# Neuronal oscillations: early biomarkers of psychiatric disease?

**DOI:** 10.3389/fnbeh.2022.1038981

**Published:** 2022-12-19

**Authors:** Anne Günther, Ileana L. Hanganu-Opatz

**Affiliations:** Institute of Developmental Neurophysiology, Center for Molecular Neurobiology, University Medical Center Hamburg-Eppendorf, Hamburg, Germany

**Keywords:** biomarkers, schizophrenia, autism, development, neuronal oscillations

## Abstract

Our understanding of the environmental and genetic factors contributing to the wide spectrum of neuropsychiatric disorders has significantly increased in recent years. Impairment of neuronal network activity during early development has been suggested as a contributor to the emergence of neuropsychiatric pathologies later in life. Still, the neurobiological substrates underlying these disorders remain yet to be fully understood and the lack of biomarkers for early diagnosis has impeded research into curative treatment options. Here, we briefly review current knowledge on potential biomarkers for emerging neuropsychiatric disease. Moreover, we summarize recent findings on aberrant activity patterns in the context of psychiatric disease, with a particular focus on their potential as early biomarkers of neuropathologies, an essential step towards pre-symptomatic diagnosis and, thus, early intervention.

## Introduction

Neurodevelopmental disorders (NDDs) are highly prevalent diseases that affect about 3% of the population worldwide. They include disorders such as schizophrenia (SCZ), some forms of epilepsy, autism spectrum disorder (ASD), and attention deficit-hyperactivity disorder (ADHD). NDDs are characterized by a wide range of symptoms, including impairments in cognition, language, emotional processing, and motor functions (Thapar et al., 2017; Savatt and Myers, 2021). Despite the devastating individual and societal burden of NDDs, no disease-modifying treatments are available, as current treatments are solely aimed to mitigate symptoms. While symptomatic treatments can alleviate some of the burdens for affected individuals, currently available treatments are only effective for some patients, only target some of the described symptoms of NDDs, and tend to have strong side-effects (Genovese and Butler, 2020; Goff, 2021).

The etiology of NDDs is multifaceted, including risk factors from genetic, epigenetic, as well as environmental sources, such as maternal immune activation during embryonal development or stress later in life (van Os and Kapur, 2009; Schmitt et al., 2014; Willsey and State, 2015; Han et al., 2021; Reichard and Zimmer-Bensch, 2021). However, despite the discovery of many new risk factors over the years, no comprehensive model of the pathophysiological mechanisms underlying NDDs has been described.

In recent years, there has been a growing understanding that developmental trajectories define the physiological and pathophysiological development of neuronal circuits (Marín, 2016). Thus, the notion of early therapeutic intervention during sensitive phases of development has gained traction in the context of potentially rescuing pathophysiological trajectories of development towards a healthy state (Veenstra-VanderWeele and Warren, 2015; Dehorter and Del Pino, 2020). However, early diagnosis of at-risk individuals before the onset of symptoms remains challenging, not only due to heterogeneity of NDDs but also due to their high levels of comorbidity, e.g., for some forms of epilepsy co-occurring with intellectual disability or ADHD co-occurring with ASD (Thapar et al., 2017). Thus, the identification of biomarkers for NDDs is essential in order to facilitate early diagnosis and, consequently, research into potential avenues of treatment.

Recent studies identified neuronal activity patterns as a potential parameter for the pre-symptomatic diagnosis of NDDs. Activity patterns were shown to be altered in patients of NDDs in a frequency-specific manner (Newson and Thiagarajan, 2019). Promisingly, in animal models of NDDs similar aberrations have been reported not only in adult animals but also during early development (Sigurdsson et al., 2010; Richter et al., 2019; Chini et al., 2020). This review focuses on early biomarkers of neuropsychiatric disease. We provide a brief overview of different neurodevelopmental psychopathologies, with a focus on two of the most prevalent NDDs, schizophrenia (SCZ) and autism spectrum disorders (ASD). Moreover, we include a summary of the current knowledge on potential biomarkers for emerging neuropsychiatric disease, before giving a brief overview of normal and aberrant activity in developing neuronal networks. A particular focus will be on relating observations from human studies to recent insights gained from animal models. Finally, we identify open questions and future research directions for linking early dysfunction of neuronal circuits with emerging disease.

## Schizophrenia

SCZ is a severe neuropsychiatric disorder with onset during young adulthood. SCZ is characterized by chronic, debilitating symptoms, which have been classified as “positive” (e.g., hallucinations, delusions), “disorganized” (e.g., formal thought disorder), and “negative” (e.g., social withdrawal, anhedonia), as well as by cognitive deficits (Jauhar et al., 2022). Despite decades of research, the pathophysiological mechanisms underlying SCZ are not entirely understood. The etiology of SCZ is heterogeneous, including environmental, epigenetic, as well as genetic risk factors, the latter ranging from chromosomal aberrations to single point mutations (van Os and Kapur, 2009). A known major risk factor for SCZ is the 22q11.2 microdeletion, which, on top of cognitive impairments, often includes metabolic burdens for affected individuals (McDonald-McGinn et al., 2015). Another risk factor for NDDs, including SCZ, is disruption of DISC1 (disrupted-in-schizophrenia 1) which is involved in coordinating neuronal development (Millar et al., 2001). Additionally, environmental insults, such as maternal immune activation during pregnancy, hypoxia, and stress (Schmitt et al., 2014), alone or in combination with genetic abnormalities, are thought to contribute to the emergence of SCZ symptoms.

Treatment options for SCZ are limited to symptomatic treatment, despite the fact that many patients respond poorly to available treatments (Goff, 2021). Additionally, most therapeutic strategies solely target “positive” symptoms of schizophrenia, whereas “negative” symptoms are unaffected (Owen et al., 2016). Crucially, pre-symptomatic diagnosis of SCZ remains challenging, due to its heterogeneous etiology and the late emergence of diverse symptoms (Jauhar et al., 2022). Notably, the first symptomatic onset of SCZ tends to be preceded by a prodromal period of up to 5 years with subtle alterations in behavior and cognition, described as “basic symptoms” (e.g., thought processing and attention) and “ultra-high-risk” symptoms (e.g., brief limited intermittent psychotic episode; Klosterkötter et al., 2001; Fusar-Poli et al., 2013). Accordingly, recent studies have focused on the prodromal period, aiming to identify reliable markers of emerging psychosis (Bernardini et al., 2017; Mikanmaa et al., 2019).

## Autism Spectrum Disorder

ASD comprises a heterogeneous group of NDDs, which are characterized by symptoms of impaired social interaction, restricted interests, and repetitive behaviors (Lord et al., 2020). The onset of ASD occurs at an early age, usually prior to the age of 3 years, and it has a prevalence of more than 1% of the total population. Similar to SCZ, the etiology of ASD is heterogeneous and risk factors include genetic aberrations as well as environmental or epigenetic factors (Lord et al., 2020; Savatt and Myers, 2021). ASD can be further split into syndromic (i.e., with additional symptoms associated with other neurological disorders) and non-syndromic (i.e., without additional symptoms) ASD, based on the underlying genetic aberrations. These aberrations range from heritable point mutations or chromosomal aberrations, all the way to *de novo* gene mutations, with the most common aberrations being mutations in synaptic genes, such as neuroligins and neurexins, or chromosomal aberrations, such as 15q11 duplications (Pinto et al., 2010; Iossifov et al., 2012; Singh and Eroglu, 2013). However, while a multitude of risk factors has been identified, no consistent model of the pathways underlying ASD has been described.

Symptomatic treatments for ASD are available, but they are often complicated by comorbidities with other disorders and only provide partial relief from symptoms (Genovese and Butler, 2020). Accordingly, a better understanding of the pathophysiological mechanisms underlying NDDs is essential in order to facilitate research into potential options for intervention.

## Animal Models of Neuropsychiatric Disease

Animal models are instrumental for basic and pre-clinical research of any human condition. Over the past century, animal models have provided essential insights into the manifold pathways underlying not only animal but also human physiology (Robinson et al., 2019). Non-human primates are particularly well-suited for research on neuropsychiatric disorders. They not only exhibit complex cognitive, social, and behavioral processes, but they also offer general similarity of brain structures compared to humans, such as the expanded prefrontal cortex (PFC), which is essential for higher-order processes (Chini and Hanganu-Opatz, 2021). With the discovery of new-generation gene editing tools, non-human primate models of NDDs have received increased interest in recent years (Lin et al., 2022). Insights into the pathophysiology of ASD have been obtained based on macaque NDD models, including a reduced expression of excitatory synaptic proteins, such as glutamate receptors and PSD95, in SHANK3-deficient macaques and an increased presence of GFAP-positive astrocytes in a brain region-specific manner (Liu et al., 2016; Zhao et al., 2017; Zhou et al., 2019). However, as of yet, the limited availability of models inadequately mirrors the multifaceted spectrum of human neuropsychiatric pathologies.

**Figure 1:**
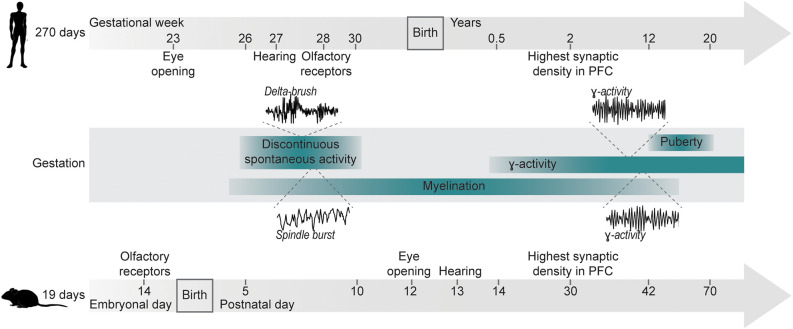
Developmental milestones for humans and mice. Developmental milestones are shown for human and murine development. Gestational durations are indicated on the left. Anatomical milestones (eye opening, development of hearing, expression of olfactory receptors) are indicated for each time line individually. Shared developmental processes (discontinuous activity, γ-activity, myelination) are indicated as age-matched ranges for both species. Insets depict examples of delta-brushes in humans [modified from Milh et al. (2007)] and spindle bursts in mice [modified from Shen and Colonnese (2016)] during early discontinuous activity, as well as filtered γ-oscillation in humans [modified from Synigal et al. (2020)] and in mice [modified from Bott et al. (2016)].

The most widely used animal models in research are rodents. Despite the evolutionary split of rats and mice from humans more than 70 million years ago, they still offer similarities in brain structure and molecular pathways of neurophysiological processes, as well as many shared developmental milestones (Figure 1). While rodent models rarely replicate the full symptomatic spectrum of any human disease, they can still mirror key features of NDDs, and targeted genetic manipulation has been used to address the contribution of individual risk factors to the pathophysiological mechanisms underlying NDDs (Diamantopoulou and Gogos, 2019).

## Biomarkers of Neuropsychiatric Disease

Biomarkers allow for clear, standardized diagnosis and monitoring of pathologies and they are crucial for research on the etiology, manifestation, and potential treatment of human disease. So far, most studies on biomarkers of neuropsychiatric disorders have focused on morphological or molecular parameters (Table 1).

**Table 1:** Biomarkers of neuropsychiatric disorders.

		**Schizophrenia**		**Autism spectrum disorder**	
**Serological markers (complement components)**	**C1q**	↑	Arakelyan et al. (2011)	↑	Corbett et al. (2007)
	**C3**	↑	Boyajyan et al. (2010)	↑	Shen et al. (2018)
		=	Arakelyan et al. (2011)		
		↓	Li et al. (2016)		
	**C4B**	↑	Maes et al. (1997)	↓	Warren et al. (1994)
		=	Schroers et al. (1997)		
	**C5**	↑	Ishii et al. (2018)	↑	Shen et al. (2018)
**Functional markers**	**EEG**		Hypofrontality at rest (Knyazeva et al., 2008); hyperfrontality during task performance (Shafritz et al., 2019)		Hypofrontality at young ages (Levin et al., 2017); hyperfrontality at later ages (Stroganova et al., 2007)
	**fMRI**		PFC hyperconnectivity during early stages (Anticevic et al., 2015)		PFC hyperconnectivity at young ages and hypoconnectivity at older ages (Jassim et al., 2021)
**Anatomical markers**	**Volume**		Reduction overall and particularly in Cg (Pantelis et al., 2003)		Increase at early stages in Th and CC, reduction at later stages (Lange et al., 2015)
**Other**			Prodromal phase of up to 5 years: behavioral and cognitive impairments (Klosterkötter et al., 2001)		

Previously identified candidates for potential biomarkers of neurodevelopmental disorders are listed. Arrows indicate an increase or reduction of serological markers, [=] indicates no detected change. PFC, prefrontal cortex; Cg, cingulate cortex; Th, thalamus; CC, corpus callosum; EEG, electroencephalogram; fMRI, functional magnetic resonance imaging.

One group of proposed NDD biomarkers are components of the microglial signaling cascade. Microglia function as the brain’s immune system and they are essential for synaptic refinement, monitoring, pruning, and maintaining synapses throughout development (Magdalon et al., 2020). Altered expression of components of the complement cascade, which underlies microglia signaling, has been reported in patients of SCZ and ASD (Warren et al., 1994; Maes et al., 1997; Schroers et al., 1997; Corbett et al., 2007; Boyajyan et al., 2010; Arakelyan et al., 2011; Li et al., 2016; Ishii et al., 2018; Shen et al., 2018; Rey et al., 2020), yet the roles of individual complement factors in the context of specific pathophysiological changes still need to be resolved (Magdalon et al., 2020; Woo et al., 2020). Interestingly, Sager et al. (2021) highlighted the relevance of age for these interactions with a change in expression of complement factors during development and a peak during early childhood between ages 3 and 5. In addition to possible molecular parameters, anatomical factors, such as volumetric assessment of gray matter, have been proposed as biomarkers of disease (Table 1). Changes in the volume of several brain regions have been reported in patients of NDDs, the most prominent change being a reduction of brain volume at later stages of these diseases (Pantelis et al., 2003; Lange et al., 2015; Hoogman et al., 2017). Additionally, fMRI data documented altered functional connectivity within networks prior to the onset of the disease, especially hyperconnectivity at early stages of SCZ as well as ASD (Anticevic et al., 2015; Jassim et al., 2021).

On the functional level, excitation/inhibition (E/I) imbalance has been proposed as a potential biomarker. E/I balance is essential for maintaining physiological network activity and disruptions of the pathways underlying excitation and/or inhibition have been described for patients with SCZ or ASD (Gao and Penzes, 2015; Sohal and Rubenstein, 2019). It is, therefore, tempting to postulate that neuronal activity patterns could provide a potential llink between genetic aberrations, physiological changes, and impaired cognitive or behavioral functions.

## Patterns of Neuronal Oscillations in Health and Disease

Precisely coordinated interactions of neurons in local and distant networks are the physiological substrate of cognitive and sensory processing, which is imperative for day-to-day survival. These network oscillations are essential for gating of incoming information (Singer, 2018) and they are conserved across species (Buzsáki et al., 2013).

Since the first electroencephalographic recordings (Berger, 1929), network oscillations within distinct frequency bands have been related to specific functions. High-frequency oscillations (gamma band, 30–100 Hz) allow for synchronization of local activity and are essential for cognitive processing, whereas slower oscillations (delta band 1–4 Hz, theta band 4–12 Hz) are more suited for coordination of distant networks, as they require less temporal precision due to the lengthened periods between alternating phases (Uhlhaas and Singer, 2013). Additionally, cross-frequency coupling between networks can mediate long-range coordination, with the phase of low-frequency oscillations in one network modulating the amplitude of high-frequency oscillations in another (Buzsáki and Draguhn, 2004).

Neuronal oscillations are known to play an essential role in cognitive processing and a link to cognitive deficits in NDDs has been postulated (Simon and Wallace, 2016; Hirano and Uhlhaas, 2021). Electroencephalographic (EEG) studies in SCZ patients have shown a general increase of power across lower-frequencies accompanied by a power decrease in the high frequency band (Newson and Thiagarajan, 2019). However, these observations depend on multiple factors, such as the task performed by the examined individuals, the stage of the disease, as well as medication. Additionally, the question of prefrontal hypo- and/or hyperactivity in NDD patients remains (Weinberger et al., 1986; Schneider et al., 2007; Stroganova et al., 2007; Knyazeva et al., 2008; Levin et al., 2017; Shafritz et al., 2019). Abnormal neuronal activity in the PFC of SCZ and ASD patients is of particular interest, as the PFC is a critical hub of cognitive functions across mammalian species (Miller, 2000). The PFC is involved in executive processes, such as attention, working memory, and decision-making, as well as social behaviors (Chini and Hanganu-Opatz, 2021), and impairments of these processes constitute the core of cognitive symptoms observed in NDDs (O’Grada and Dinan, 2007). Accordingly, many studies have focused on prefrontal function in disease and both hyper- as well as hypofrontality have been observed in SCZ and ASD patients, depending on brain state or performed task (Dawson et al., 1995; Knyazeva et al., 2008; van Diessen et al., 2015; Shafritz et al., 2019). Notably, altered prefrontal activity and connectivity have been shown in mouse models of SCZ and ASD (Fénelon et al., 2013; Crabtree et al., 2017; Wang et al., 2018). In addition, multiple studies on mouse models have identified prefrontal layer 2/3 pyramidal neurons to be particularly vulnerable to disruptions and to show structural and functional impairment, such as reduced synaptic density, in models of NDDs (Cooper and Koleske, 2014; Lazaro et al., 2019; Comer et al., 2020; Nagahama et al., 2020). Interestingly, similar alterations in spine densities have been reported in postmortem brain tissue from SCZ patients (Konopaske et al., 2014).

In SCZ and ASD patients, weaker prefrontal-hippocampal communication has been suggested to potentially underlie cognitive deficits (Meyer-Lindenberg et al., 2005; Cooper et al., 2017). Correspondingly, in mouse models of SCZ and ASD, prefrontal-hippocampal interactions are disrupted. For example, in a mouse model mirroring the human 22q11.2 microdeletion, hippocampal-prefrontal theta-frequency synchrony is decreased during working memory tasks (Sigurdsson et al., 2010). In a mouse model of ASD, manipulation of *Pogz* expression similarly affected prefrontal-hippocampal synchronization (Cunniff et al., 2020).

These consistent changes within prefrontal-hippocampal circuits across species, models, and pathologies, indicate that altered patterns of oscillatory activity and synchrony-based coupling might contribute to the emergence of cognitive deficits. Therefore, a key question to be addressed is whether aberrant activity patterns develops earlier, potentially even before symptomatic onset of the disease.

## Early Patterns of Neuronal Activity in Schizophrenia and Autism Spectrum Disorders

Precisely timed coordination of molecular and cellular processes is required in order to define developmental trajectories early in life and all the way to adulthood. In the human CNS, the ability to respond to new experiences persists throughout development, including neuronal cell migration at embryonal ages as well as neuronal differentiation, synaptogenesis, synaptic plasticity, and synaptic pruning all the way to adulthood. However, this lasting plasticity might also imply an increased vulnerability towards insults. It has been hypothesized that the trajectory of physiological development is particularly sensitive during specific phases (Larsen and Luna, 2018; Sakurai and Gamo, 2019).

Patterns of electrical activity and their temporal coordination change alongside development (Chini and Hanganu-Opatz, 2021). Spontaneous, highly synchronized activity is a hallmark of neuronal circuits during early development (Babola et al., 2018; Martini et al., 2021). This activity is discontinuous (i.e., periods of oscillatory discharges alternate with silent periods) and has been shown for all mammalian species investigated at a comparable stage of brain development (i.e., 2nd–3rd gestational trimester in humans and early postnatal in rodents; Figure 1; Khazipov et al., 2004; Hanganu et al., 2006; Colonnese et al., 2010). Subsequent desynchronization of activity mirrors the increasing influence of sensory inputs shaping neuronal activity (Toyoizumi et al., 2013). Notably, due to different types of processed information and timelines of circuit assembly, different areas and functions exhibit distinct sensitive periods of maturation, e.g., the human visual system being particularly sensitive during the first few years of life, while language acquisition is sensitive during the first year after birth (Wiesel, 1982; Daw, 1998; Anderson and Reidy, 2012; Friedmann and Rusou, 2015; Gire et al., 2022). Similarly, in rodents, experience shapes synaptic circuits of visual and auditory systems during periods of enhanced plasticity, following eye/ear opening (Lehmann and Löwel, 2008; Rinaldi Barkat et al., 2011). Even for circuits involved in cognitive processing in mice, recent data suggested the presence of time windows exhibiting increased sensitivity.

Several studies have hypothesized that therapeutic interventions at earlier stages of development might hold promise for NDD treatment. Birth cohort studies showed developmental anomalies of behavior and cognition already in 4-year-old children, who developed SCZ later in life (Welham et al., 2009). Notably, in prodromal SCZ patients, the elevation of frontal oscillatory activity in delta and theta frequency bands has been detected (van Tricht et al., 2014), although the underlying neuronal mechanisms remain poorly understood. A comprehensive summary of available data on alterations of neuronal dynamics in SCZ patients and high-risk prodromal individuals has previously been done (Mikanmaa et al., 2019).

Similarly, mouse models of NDDs have been investigated during early development. For example, immune-challenged mice with altered DISC1 expression showed disrupted patterns of network activity in beta-gamma range in the PFC during the first two postnatal weeks (Chini et al., 2020). Additionally, prefrontal-hippocampal coupling was decreased in theta frequency bands at early ages (Xu et al., 2019; Song et al., 2022). These early deficits in neuronal activity have been proposed to contribute to poor cognitive performance at adult age. Notably, prominent dysfunctions induced by transient disruption of DISC1 expression in immune-challenged mice, as well as perinatal disruption of Arc/Arg3.1 (activity-regulated cytoskeleton-associated protein) suggest the existence of specific phases during which hippocampal and prefrontal activity play a crucial role (Gao et al., 2018, 2019; Xu et al., 2019).

In summary, these findings suggest that disruption of early neuronal activity patterns relates to the emergence of NDDs later in life and might offer a promising avenue for potential treatments.

## Future Perspectives for Diagnosis and Intervention

Despite the ethical and technical challenges inherent to investigations of early human development, the data reviewed here highlight early development as a crucial time window for addressing the circuit miswiring which underlies adult cognitive burdens of neuropsychiatric disorders. Therefore, sustained efforts are necessary in order to develop tailored interventions which minimize this burden for affected individuals. Early intensive behavioral intervention (EIBI) in children with emerging ASD has shown promising results for improving adaptive behaviors later in life (Tiura et al., 2017; Reichow et al., 2018). Similarly, early intervention using psychosocial and psychopharmacological treatments immediately after the onset of psychosis has shown some promising results in SCZ patients, e.g., reduced severity of positive as well as negative symptoms (Correll et al., 2018). However, the effects of this described early intervention do not persist over time (Chan et al., 2019).

In this context, the need for reliable biomarkers of early dysfunction is evident. The wide spectrum of dynamic features inherent to neuronal oscillations might constitute a powerful tool for identifying, describing, and potentially even rerouting disorder-specific deficits. Specifically for SCZ, previous hypotheses have focused on altered neuronal activity during the prodromal period or during the time of the first episode of psychosis as a potential time window for intervention (Millan et al., 2016; Mikanmaa et al., 2019). However, the idea of sensitive phases during early development as potential points for intervention has gained traction in recent years (Dehorter and Del Pino, 2020). Particularly the roles of GABAergic signaling as well as of fast-spiking parvalbumin (PV) interneurons have been highlighted as critical modulators of sensitive periods during development (Marín, 2016). Notably, more than a decade ago, PV interneuron dysfunction was linked to the characteristic cognitive deficits of SCZ (Lewis et al., 2012).

Additionally, identifying developmental phases of aberrant activity in NDDs is a prerequisite for the rescue of physiological trajectories during development. Recently, manipulation of activity in a specific subset of prefrontal pyramidal neurons in young mice was shown to alter developmental trajectories and to have long-term effects on brain function and cognitive processes (Bitzenhofer et al., 2021). Thus, identifying aberrant patterns of early network oscillations might not only provide an approach for pre-symptomatic diagnosis but also facilitate a better understanding of NDDs. In humans, approaches for direct modulation of neuronal oscillations (e.g., transcranial alternating current stimulation) have been used in a frequency-specific manner (Fröhlich et al., 2015; Jones et al., 2020), revealing promising tools for the treatment of psychiatric disorders (Allenby et al., 2018; Ahn et al., 2019).

In conclusion, monitoring early neuronal oscillations is highly instrumental for understanding physiological and pathophysiological development. Early aberrations of neuronal activity patterns could serve as biomarkers of NDDs and thereby open up new avenues for non-invasive, pre-symptomatic diagnosis of at-risk patients, as well as provide potential tools for the directed treatment of emerging neuropsychiatric disease.

## Author Contributions

AG and IH-O drafted, wrote, and edited the manuscript. All authors contributed to the article and approved the submitted version.

## References

[B1] Ahn S. Mellin J. M. Alagapan S. Alexander M. L. Gilmore J. H. Jarskog L. F.. (2019). Targeting reduced neural oscillations in patients with schizophrenia by transcranial alternating current stimulation. Neuroimage 186, 126–136. 10.1016/j.neuroimage.2018.10.056 30367952 PMC6338501

[B2] Allenby C. Falcone M. Bernardo L. Wileyto P. Rostain A. Ramsay J. R.. (2018). Transcranial direct current brain stimulation decreases impulsivity in ADHD. Brain Stimul. 11, 974–981. 10.1016/j.brs.2018.04.016 29885858 PMC6109423

[B3] Anderson P. J. Reidy N. (2012). Assessing executive function in preschoolers. Neuropsychol. Rev. 22, 345–360. 10.1007/s11065-012-9220-3 23109046

[B4] Anticevic A. Hu X. Xiao Y. Hu J. Li F. Bi F.. (2015). Early-course unmedicated schizophrenia patients exhibit elevated prefrontal connectivity associated with longitudinal change. J. Neurosci. 35, 267–286. 10.1523/JNEUROSCI.2310-14.2015 25568120 PMC4287147

[B5] Arakelyan A. Zakharyan R. Khoyetsyan A. Poghosyan D. Aroutiounian R. Mrazek F.. (2011). Functional characterization of the complement receptor type 1 and its circulating ligands in patients with schizophrenia. BMC Clin. Pathol. 11:10. 10.1186/1472-6890-11-10 21867543 PMC3176470

[B6] Babola T. A. Li S. Gribizis A. Lee B. J. Issa J. B. Wang H. C.. (2018). Homeostatic control of spontaneous activity in the developing auditory system. Neuron 99, 511–524.e5. 10.1016/j.neuron.2018.07.004 30077356 PMC6100752

[B7] Berger H. (1929). Über das Elektrenkephalogramm des Menschen. Arch. F. Psychiatr. Nervenkrankh. 87, 527–570. 10.1007/BF01797193

[B8] Bernardini F. Attademo L. Cleary S. D. Luther C. Shim R. S. Quartesan R.. (2017). Risk prediction models in psychiatry: toward a new frontier for the prevention of mental illnesses. J. Clin. Psychiatry 78, 572–583. 10.4088/JCP.15r10003 27337225

[B9] Bitzenhofer S. H. Pöpplau J. A. Chini M. Marquardt A. Hanganu-Opatz I. L. (2021). A transient developmental increase in prefrontal activity alters network maturation and causes cognitive dysfunction in adult mice. Neuron 109, 1350–1364.e6. 10.1016/j.neuron.2021.02.011 33675685 PMC8063718

[B10] Bott J.-B. Muller M.-A. Jackson J. Aubert J. Cassel J.-C. Mathis C.. (2016). Spatial reference memory is associated with modulation of theta-gamma coupling in the dentate gyrus. Cereb. Cortex 26, 3744–3753. 10.1093/cercor/bhv177 26250776

[B11] Boyajyan A. Khoyetsyan A. Chavushyan A. (2010). Alternative complement pathway in schizophrenia. Neurochem. Res. 35, 894–898. 10.1007/s11064-010-0126-2 20101522

[B12] Buzsáki G. Draguhn A. (2004). Neuronal oscillations in cortical networks. Science 304, 1926–1929. 10.1126/science.1099745 15218136

[B13] Buzsáki G. Logothetis N. Singer W. (2013). Scaling brain size, keeping timing: evolutionary preservation of brain rhythms. Neuron 80, 751–764. 10.1016/j.neuron.2013.10.002 24183025 PMC4009705

[B14] Chan S. K. W. Chan H. Y. V. Devlin J. Bastiampillai T. Mohan T. Hui C. L. M.. (2019). A systematic review of long-term outcomes of patients with psychosis who received early intervention services. Int. Rev. Psychiatry 31, 425–440. 10.1080/09540261.2019.1643704 31353981

[B15] Chini M. Hanganu-Opatz I. L. (2021). Prefrontal cortex development in health and disease: lessons from rodents and humans. Trends Neurosci. 44, 227–240. 10.1016/j.tins.2020.10.017 33246578

[B16] Chini M. Pöpplau J. A. Lindemann C. Carol-Perdiguer L. Hnida M. Oberländer V.. (2020). Resolving and rescuing developmental miswiring in a mouse model of cognitive impairment. Neuron 105, 60–74.e7. 10.1016/j.neuron.2019.09.042 31733940 PMC6953432

[B17] Colonnese M. T. Kaminska A. Minlebaev M. Milh M. Bloem B. Lescure S.. (2010). A conserved switch in sensory processing prepares developing neocortex for vision. Neuron 67, 480–498. 10.1016/j.neuron.2010.07.015 20696384 PMC2946625

[B18] Comer A. L. Jinadasa T. Sriram B. Phadke R. A. Kretsge L. N. Nguyen T. P. H.. (2020). Increased expression of schizophrenia-associated gene C4 leads to hypoconnectivity of prefrontal cortex and reduced social interaction. PLoS Biol. 18:e3000604. 10.1371/journal.pbio.3000604 31935214 PMC6959572

[B19] Cooper M. A. Koleske A. J. (2014). Ablation of ErbB4 from excitatory neurons leads to reduced dendritic spine density in mouse prefrontal cortex. J. Comp. Neurol. 522, 3351–3362. 10.1002/cne.23615 24752666 PMC4107058

[B20] Cooper R. A. Richter F. R. Bays P. M. Plaisted-Grant K. C. Baron-Cohen S. Simons J. S. (2017). Reduced hippocampal functional connectivity during episodic memory retrieval in autism. Cereb. Cortex 27, 888–902. 10.1093/cercor/bhw417 28057726 PMC5390398

[B21] Corbett B. A. Kantor A. B. Schulman H. Walker W. L. Lit L. Ashwood P.. (2007). A proteomic study of serum from children with autism showing differential expression of apolipoproteins and complement proteins. Mol. Psychiatry 12, 292–306. 10.1038/sj.mp.4001943 17189958

[B22] Correll C. U. Galling B. Pawar A. Krivko A. Bonetto C. Ruggeri M.. (2018). Comparison of early intervention services vs treatment as usual for early-phase psychosis. JAMA Psychiatry 75, 555–565. 10.1001/jamapsychiatry.2018.0623 29800949 PMC6137532

[B23] Crabtree G. W. Sun Z. Kvajo M. Broek J. A. C. Fénelon K. McKellar H.. (2017). Alteration of neuronal excitability and short-term synaptic plasticity in the prefrontal cortex of a mouse model of mental illness. J. Neurosci. 37, 4158–4180. 10.1523/JNEUROSCI.4345-15.2017 28283561 PMC5391686

[B24] Cunniff M. M. Markenscoff-Papadimitriou E. Ostrowski J. Rubenstein J. L. Sohal V. S. (2020). Altered hippocampal-prefrontal communication during anxiety-related avoidance in mice deficient for the autism-associated gene Pogz. eLife 9:e54835. 10.7554/eLife.54835 33155545 PMC7682992

[B25] Daw N. W. (1998). Critical periods and amblyopi. Arch. Ophthalmol. 116, 502–505. 10.1001/archopht.116.4.502 9565050

[B26] Dawson G. Klinger L. G. Panagiotides H. Lewy A. Castelloe P. (1995). Subgroups of autistic children based on social behavior display distinct patterns of brain activity. J. Abnorm. Child Psychol. 23, 569–583. 10.1007/BF01447662 8568080

[B27] Dehorter N. Del Pino I. (2020). Shifting developmental trajectories during critical periods of brain formation. Front. Cell. Neurosci. 14:283. 10.3389/fncel.2020.00283 33132842 PMC7513795

[B28] Diamantopoulou A. Gogos J. A. (2019). Neurocognitive and perceptual processing in genetic mouse models of schizophrenia: emerging lessons. Neuroscientist 25, 597–619. 10.1177/1073858418819435 30654694

[B29] Fénelon K. Xu B. Lai C. S. Mukai J. Markx S. Stark K. L.. (2013). The pattern of cortical dysfunction in a mouse model of a schizophrenia-related microdeletion. J. Neurosci. 33, 14825–14839. 10.1523/JNEUROSCI.1611-13.2013 24027283 PMC3771024

[B30] Friedmann N. Rusou D. (2015). Critical period for first language: the crucial role of language input during the first year of life. Curr. Opin. Neurobiol. 35, 27–34. 10.1016/j.conb.2015.06.003 26111432

[B31] Fröhlich F. Sellers K. K. Cordle A. L. (2015). Targeting the neurophysiology of cognitive systems with transcranial alternating current stimulation (tACS). Expert Rev. Neurother. 15, 145–167. 10.1586/14737175.2015.992782 25547149 PMC4634940

[B32] Fusar-Poli P. Borgwardt S. Bechdolf A. Addington J. Riecher-Rössler A. Schultze-Lutter F.. (2013). The psychosis high-risk state. JAMA Psychiatry 70, 107–120. 10.1001/jamapsychiatry.2013.269 23165428 PMC4356506

[B34] Gao X. Castro-Gomez S. Grendel J. Graf S. Süsens U. Binkle L.. (2018). Arc/Arg3.1 mediates a critical period for spatial learning and hippocampal networks. Proc. Natl. Acad. Sci. U S A 115, 12531–12536. 10.1073/pnas.1810125115 30442670 PMC6298089

[B35] Gao X. Grendel J. Muhia M. Castro-Gomez S. Süsens U. Isbrandt D.. (2019). Disturbed prefrontal cortex activity in the absence of schizophrenia-like behavioral dysfunction in Arc/Arg3.1 deficient mice. J. Neurosci. 39, 8149–8163. 10.1523/JNEUROSCI.0623-19.2019 31488612 PMC6786822

[B33] Gao R. Penzes P. (2015). Common mechanisms of excitatory and inhibitory imbalance in schizophrenia and autism spectrum disorders. Curr. Mol. Med. 15, 146–167. 10.2174/1566524015666150303003028 25732149 PMC4721588

[B36] Genovese A. Butler M. G. (2020). Clinical assessment, genetics and treatment approaches in autism spectrum disorder (ASD). Int. J. Mol. Sci. 21:4726. 10.3390/ijms21134726 32630718 PMC7369758

[B37] Gire C. Garbi A. Zahed M. Beltran Anzola A. Tosello B. Datin-Dorrière V. (2022). Neurobehavioral phenotype and dysexecutive syndrome of preterm children: comorbidity or trigger? An update. Children (Basel) 9:239. 10.3390/children9020239 35204960 PMC8870742

[B38] Goff D. C. (2021). The pharmacologic treatment of schizophrenia—2021. JAMA 325, 175–176. 10.1001/jama.2020.19048 33369626

[B39] Han V. X. Patel S. Jones H. F. Dale R. C. (2021). Maternal immune activation and neuroinflammation in human neurodevelopmental disorders. Nat. Rev. Neurol. 17, 564–579. 10.1038/s41582-021-00530-8 34341569

[B40] Hanganu I. L. Ben-Ari Y. Khazipov R. (2006). Retinal waves trigger spindle bursts in the neonatal rat visual cortex. J. Neurosci. 26, 6728–6736. 10.1523/JNEUROSCI.0752-06.2006 16793880 PMC6673818

[B41] Hirano Y. Uhlhaas P. J. (2021). Current findings and perspectives on aberrant neural oscillations in schizophrenia. Psychiatry Clin. Neurosci. 75, 358–368. 10.1111/pcn.13300 34558155

[B42] Hoogman M. Bralten J. Hibar D. P. Mennes M. Zwiers M. P. Schweren L.. (2017). Subcortical brain volume differences of participants with ADHD across the lifespan: an ENIGMA collaboration. Lancet Psychiatry 4, 310–319. 10.1016/S2215-0366(17)30049-4 28219628 PMC5933934

[B43] Iossifov I. Ronemus M. Levy D. Wang Z. Hakker I. Rosenbaum J.. (2012). *De novo* gene disruptions in children on the autistic spectrum. Neuron 74, 285–299. 10.1016/j.neuron.2012.04.009 22542183 PMC3619976

[B44] Ishii T. Hattori K. Miyakawa T. Watanabe K. Hidese S. Sasayama D.. (2018). Increased cerebrospinal fluid complement C5 levels in major depressive disorder and schizophrenia. Biochem. Biophys. Res. Commun. 497, 683–688. 10.1016/j.bbrc.2018.02.131 29454970

[B45] Jassim N. Baron-Cohen S. Suckling J. (2021). Meta-analytic evidence of differential prefrontal and early sensory cortex activity during non-social sensory perception in autism. Neurosci. Biobehav. Rev. 127, 146–157. 10.1016/j.neubiorev.2021.04.014 33887326

[B46] Jauhar S. Johnstone M. McKenna P. J. (2022). Schizophrenia. Lancet 399, 473–486. 10.1016/S0140-6736(21)01730-X 35093231

[B47] Jones K. T. Johnson E. L. Tauxe Z. S. Rojas D. C. (2020). Modulation of auditory gamma-band responses using transcranial electrical stimulation. J. Neurophysiol. 123, 2504–2514. 10.1152/jn.00003.2020 32459551

[B48] Khazipov R. Sirota A. Leinekugel X. Holmes G. L. Ben-Ari Y. Buzsáki G. (2004). Early motor activity drives spindle bursts in the developing somatosensory cortex. Nature 432, 758–761. 10.1038/nature03132 15592414

[B49] Klosterkötter J. Hellmich M. Steinmeyer E. M. Schultze-Lutter F. (2001). Diagnosing schizophrenia in the initial prodromal phase. Arch. Gen. Psychiatry 58, 158–164. 10.1001/archpsyc.58.2.158 11177117

[B50] Knyazeva M. G. Jalili M. Meuli R. Hasler M. De Feo O. Do K. Q. (2008). Alpha rhythm and hypofrontality in schizophrenia. Acta Psychiatr. Scand. 118, 188–199. 10.1111/j.1600-0447.2008.01227.x 18636993

[B51] Konopaske G. T. Lange N. Coyle J. T. Benes F. M. (2014). Prefrontal cortical dendritic spine pathology in schizophrenia and bipolar disorder. JAMA Psychiatry 71, 1323–1331. 10.1001/jamapsychiatry.2014.1582 25271938 PMC5510541

[B52] Lange N. Travers B. G. Bigler E. D. Prigge M. B. D. Froehlich A. L. Nielsen J. A.. (2015). Longitudinal volumetric brain changes in autism spectrum disorder ages 6-35 years. Autism Res. 8, 82–93. 10.1002/aur.1427 25381736 PMC4344386

[B53] Larsen B. Luna B. (2018). Adolescence as a neurobiological critical period for the development of higher-order cognition. Neurosci. Biobehav. Rev. 94, 179–195. 10.1016/j.neubiorev.2018.09.005 30201220 PMC6526538

[B54] Lazaro M. T. Taxidis J. Shuman T. Bachmutsky I. Ikrar T. Santos R.. (2019). Reduced prefrontal synaptic connectivity and disturbed oscillatory population dynamics in the CNTNAP2 model of autism. Cell Rep. 27, 2567–2578.e6. 10.1016/j.celrep.2019.05.006 31141683 PMC6553483

[B55] Lehmann K. Löwel S. (2008). Age-dependent ocular dominance plasticity in adult mice. PLoS One 3:e3120. 10.1371/journal.pone.0003120 18769674 PMC2518841

[B56] Levin A. R. Varcin K. J. O’Leary H. M. Tager-Flusberg H. Nelson C. A. (2017). EEG power at 3 months in infants at high familial risk for autism. J. Neurodev. Disord. 9:34. 10.1186/s11689-017-9214-9 28903722 PMC5598007

[B57] Lewis D. A. Curley A. A. Glausier J. R. Volk D. W. (2012). Cortical parvalbumin interneurons and cognitive dysfunction in schizophrenia. Trends Neurosci. 35, 57–67. 10.1016/j.tins.2011.10.004 22154068 PMC3253230

[B58] Li H. Zhang Q. Li N. Wang F. Xiang H. Zhang Z.. (2016). Plasma levels of Th17-related cytokines and complement C3 correlated with aggressive behavior in patients with schizophrenia. Psychiatry Res. 246, 700–706. 10.1016/j.psychres.2016.10.061 27829509

[B59] Lin Y. Li J. Li C. Tu Z. Li S. Li X.-J.. (2022). Application of CRISPR/Cas9 system in establishing large animal models. Front. Cell Dev. Biol. 10:919155. 10.3389/fcell.2022.919155 35656550 PMC9152178

[B60] Liu Z. Li X. Zhang J.-T. Cai Y.-J. Cheng T.-L. Cheng C.. (2016). Autism-like behaviours and germline transmission in transgenic monkeys overexpressing MeCP2. Nature 530, 98–102. 10.1038/nature16533 26808898

[B61] Lord C. Brugha T. S. Charman T. Cusack J. Dumas G. Frazier T.. (2020). Autism spectrum disorder. Nat. Rev. Dis. Primer 6:5. 10.1038/s41572-019-0138-4 31949163 PMC8900942

[B62] Maes M. Delange J. Ranjan R. Meltzer H. Y. Desnyder R. Cooremans W.. (1997). Acute phase proteins in schizophrenia, mania and major depression: modulation by psychotropic drugs. Psychiatry Res. 66, 1–11. 10.1016/s0165-1781(96)02915-0 9061799

[B63] Magdalon J. Mansur F. Teles e Silva A. L. de Goes V. A. Reiner O. Sertié A. L. (2020). Complement system in brain architecture and neurodevelopmental disorders. Front. Neurosci. 14:23. 10.3389/fnins.2020.00023 32116493 PMC7015047

[B64] Marín O. (2016). Developmental timing and critical windows for the treatment of psychiatric disorders. Nat. Med. 22, 1229–1238. 10.1038/nm.4225 27783067

[B65] Martini F. J. Guillamón-Vivancos T. Moreno-Juan V. Valdeolmillos M. López-Bendito G. (2021). Spontaneous activity in developing thalamic and cortical sensory networks. Neuron 109, 2519–2534. 10.1016/j.neuron.2021.06.026 34293296 PMC7611560

[B66] McDonald-McGinn D. M. Sullivan K. E. Marino B. Philip N. Swillen A. Vorstman J. A. S.. (2015). 22q11.2 deletion syndrome. Nat. Rev. Dis. Primer 1:15071. 10.1038/nrdp.2015.71 27189754 PMC4900471

[B67] Meyer-Lindenberg A. S. Olsen R. K. Kohn P. D. Brown T. Egan M. F. Weinberger D. R.. (2005). Regionally specific disturbance of dorsolateral prefrontal-hippocampal functional connectivity in schizophrenia. Arch. Gen. Psychiatry 62, 379–386. 10.1001/archpsyc.62.4.379 15809405

[B68] Mikanmaa E. Grent-’t-Jong T. Hua L. Recasens M. Thune H. Uhlhaas P. J. (2019). Towards a neurodynamical understanding of the prodrome in schizophrenia. Neuroimage 190, 144–153. 10.1016/j.neuroimage.2017.11.026 29175199

[B69] Milh M. Kaminska A. Huon C. Lapillonne A. Ben-Ari Y. Khazipov R. (2007). Rapid cortical oscillations and early motor activity in premature human neonate. Cereb. Cortex 17, 1582–1594. 10.1093/cercor/bhl069 16950867

[B70] Millan M. J. Andrieux A. Bartzokis G. Cadenhead K. Dazzan P. Fusar-Poli P.. (2016). Altering the course of schizophrenia: progress and perspectives. Nat. Rev. Drug Discov. 15, 485–515. 10.1038/nrd.2016.28 26939910

[B71] Millar J. K. Christie S. Anderson S. Lawson D. Loh D. H.-W. Devon R. S.. (2001). Genomic structure and localisation within a linkage hotspot of disrupted in schizophrenia 1, a gene disrupted by a translocation segregating with schizophrenia. Mol. Psychiatry 6, 173–178. 10.1038/sj.mp.4000784 11317219

[B72] Miller E. K. (2000). The prefontral cortex and cognitive control. Nat. Rev. Neurosci. 1, 59–65. 10.1038/35036228 11252769

[B73] Nagahama K. Sakoori K. Watanabe T. Kishi Y. Kawaji K. Koebis M.. (2020). Setd1a insufficiency in mice attenuates excitatory synaptic function and recapitulates schizophrenia-related behavioral abnormalities. Cell Rep. 32:108126. 10.1016/j.celrep.2020.108126 32937141

[B74] Newson J. J. Thiagarajan T. C. (2019). EEG frequency bands in psychiatric disorders: a review of resting state studies. Front. Hum. Neurosci. 12:521. 10.3389/fnhum.2018.00521 30687041 PMC6333694

[B75] O’Grada C. Dinan T. (2007). Executive function in schizophrenia: what impact do antipsychotics have? Hum. Psychopharmacol. 22, 397–406. 10.1002/hup.861 17579928

[B76] Owen M. J. Sawa A. Mortensen P. B. (2016). Schizophrenia. Lancet 388, 86–97. 10.1016/S0140-6736(15)01121-6 26777917 PMC4940219

[B77] Pantelis C. Velakoulis D. McGorry P. D. Wood S. J. Suckling J. Phillips L. J.. (2003). Neuroanatomical abnormalities before and after onset of psychosis: a cross-sectional and longitudinal MRI comparison. Lancet 361, 281–288. 10.1016/S0140-6736(03)12323-9 12559861

[B78] Pinto D. Pagnamenta A. T. Klei L. Anney R. Merico D. Regan R.. (2010). Functional impact of global rare copy number variation in autism spectrum disorders. Nature 466, 368–372. 10.1038/nature09146 20531469 PMC3021798

[B79] Reichard J. Zimmer-Bensch G. (2021). The epigenome in neurodevelopmental disorders. Front. Neurosci. 15:776809. 10.3389/fnins.2021.776809 34803599 PMC8595945

[B80] Reichow B. Hume K. Barton E. E. Boyd B. A. (2018). Early intensive behavioral intervention (EIBI) for young children with autism spectrum disorders (ASD). Cochrane Database Syst. Rev. 5:CD009260. 10.1002/14651858.CD009260.pub3 29742275 PMC6494600

[B81] Rey R. Suaud-Chagny M.-F. Bohec A.-L. Dorey J.-M. d’Amato T. Tamouza R.. (2020). Overexpression of complement component C4 in the dorsolateral prefrontal cortex, parietal cortex, superior temporal gyrus and associative striatum of patients with schizophrenia. Brain Behav. Immun. 90, 216–225. 10.1016/j.bbi.2020.08.019 32827700

[B82] Richter M. Murtaza N. Scharrenberg R. White S. H. Johanns O. Walker S.. (2019). Altered TAOK2 activity causes autism-related neurodevelopmental and cognitive abnormalities through RhoA signaling. Mol. Psychiatry 24, 1329–1350. 10.1038/s41380-018-0025-5 29467497 PMC6756231

[B83] Rinaldi Barkat T. Polley D. B. Hensch T. K. (2011). A critical period for auditory thalamocortical connectivity. Nat. Neurosci. 14, 1189–1194. 10.1038/nn.2882 21804538 PMC3419581

[B84] Robinson N. B. Krieger K. Khan F. M. Huffman W. Chang M. Naik A.. (2019). The current state of animal models in research: a review. Int. J. Surg. 72, 9–13. 10.1016/j.ijsu.2019.10.015 31627013

[B85] Sager R. E. H. Walker A. K. Middleton F. Robinson K. Webster M. J. Weickert C. S. (2021). Trajectory of change in brain complement factors from neonatal to young adult humans. J. Neurochem. 157, 479–493. 10.1111/jnc.15241 33190236

[B86] Sakurai T. Gamo N. J. (2019). Cognitive functions associated with developing prefrontal cortex during adolescence and developmental neuropsychiatric disorders. Neurobiol. Dis. 131:104322. 10.1016/j.nbd.2018.11.007 30423472

[B87] Savatt J. M. Myers S. M. (2021). Genetic testing in neurodevelopmental disorders. Front. Pediatr. 9:526779. 10.3389/fped.2021.526779 33681094 PMC7933797

[B88] Schmitt A. Malchow B. Hasan A. Falkai P. (2014). The impact of environmental factors in severe psychiatric disorders. Front. Neurosci. 8:19. 10.3389/fnins.2014.00019 24574956 PMC3920481

[B89] Schneider F. Habel U. Reske M. Kellermann T. Stöcker T. Shah N. J.. (2007). Neural correlates of working memory dysfunction in first-episode schizophrenia patients: an fMRI multi-center study. Schizophr. Res. 89, 198–210. 10.1016/j.schres.2006.07.021 17010573

[B90] Schroers R. Nöthen M. M. Rietschel M. Albus M. Maier W. Schwab S.. (1997). Investigation of complement C4B deficiency in schizophrenia. Hum. Hered. 47, 279–282. 10.1159/000154424 9358016

[B91] Shafritz K. M. Ikuta T. Greene A. Robinson D. G. Gallego J. Lencz T.. (2019). Frontal lobe functioning during a simple response conflict task in first-episode psychosis and its relationship to treatment response. Brain Imaging Behav. 13, 541–553. 10.1007/s11682-018-9876-2 29744804 PMC6226360

[B92] Shen J. Colonnese M. T. (2016). Development of activity in the mouse visual cortex. J. Neurosci. 36, 12259–12275. 10.1523/JNEUROSCI.1903-16.2016 27903733 PMC5148222

[B93] Shen L. Zhang K. Feng C. Chen Y. Li S. Iqbal J.. (2018). iTRAQ-based proteomic analysis reveals protein profile in plasma from children with autism. Proteomics Clin. Appl. 12:e1700085. 10.1002/prca.201700085 29274201

[B94] Sigurdsson T. Stark K. L. Karayiorgou M. Gogos J. A. Gordon J. A. (2010). Impaired hippocampal-prefrontal synchrony in a genetic mouse model of schizophrenia. Nature 464, 763–767. 10.1038/nature08855 20360742 PMC2864584

[B95] Simon D. M. Wallace M. T. (2016). Dysfunction of sensory oscillations in autism spectrum disorder. Neurosci. Biobehav. Rev. 68, 848–861. 10.1016/j.neubiorev.2016.07.016 27451342 PMC5119455

[B96] Singer W. (2018). Neuronal oscillations: unavoidable and useful? Eur. J. Neurosci. 48, 2389–2398. 10.1111/ejn.13796 29247490

[B97] Singh S. K. Eroglu C. (2013). Neuroligins provide molecular links between syndromic and non-syndromic autism. Sci. Signal. 6:re4. 10.1126/scisignal.2004102 23838185 PMC4000534

[B98] Sohal V. S. Rubenstein J. L. R. (2019). Excitation-inhibition balance as a framework for investigating mechanisms in neuropsychiatric disorders. Mol. Psychiatry 24, 1248–1257. 10.1038/s41380-019-0426-0 31089192 PMC6742424

[B99] Song L. Xu X. Putthoff P. Fleck D. Spehr M. Hanganu-Opatz I. L. (2022). Sparser and less efficient hippocampal-prefrontal projections account for developmental network dysfunction in a model of psychiatric risk mediated by gene-environment interaction. J. Neurosci. 42, 601–618. 10.1523/JNEUROSCI.1203-21.2021 34844990 PMC8805616

[B100] Stroganova T. A. Nygren G. Tsetlin M. M. Posikera I. N. Gillberg C. Elam M.. (2007). Abnormal EEG lateralization in boys with autism. Clin. Neurophysiol. 118, 1842–1854. 10.1016/j.clinph.2007.05.005 17581774

[B101] Synigal S. R. Teoh E. S. Lalor E. C. (2020). Including measures of high gamma power can improve the decoding of natural speech from EEG. Front. Hum. Neurosci. 14:130. 10.3389/fnhum.2020.00130 32410969 PMC7200998

[B102] Thapar A. Cooper M. Rutter M. (2017). Neurodevelopmental disorders. Lancet Psychiatry 4, 339–346. 10.1016/S2215-0366(16)30376-5 27979720

[B103] Tiura M. Kim J. Detmers D. Baldi H. (2017). Predictors of longitudinal ABA treatment outcomes for children with autism: a growth curve analysis. Res. Dev. Disabil. 70, 185–197. 10.1016/j.ridd.2017.09.008 28963874

[B104] Toyoizumi T. Miyamoto H. Yazaki-Sugiyama Y. Atapour N. Hensch T. K. Miller K. D. (2013). A theory of the transition to critical period plasticity: inhibition selectively suppresses spontaneous activity. Neuron 80, 51–63. 10.1016/j.neuron.2013.07.022 24094102 PMC3800182

[B105] Uhlhaas P. J. Singer W. (2013). High-frequency oscillations and the neurobiology of schizophrenia. Dialogues Clin. Neurosci. 15, 301–313. 10.31887/DCNS.2013.15.3/puhlhaas 24174902 PMC3811102

[B106] van Diessen E. Senders J. Jansen F. E. Boersma M. Bruining H. (2015). Increased power of resting-state gamma oscillations in autism spectrum disorder detected by routine electroencephalography. Eur. Arch. Psychiatry Clin. Neurosci. 265, 537–540. 10.1007/s00406-014-0527-3 25182536

[B107] van Os J. Kapur S. (2009). Schizophrenia. Lancet 374, 635–645. 10.1016/S0140-6736(09)60995-8 19700006

[B108] van Tricht M. J. Ruhrmann S. Arns M. Müller R. Bodatsch M. Velthorst E.. (2014). Can quantitative EEG measures predict clinical outcome in subjects at Clinical High Risk for psychosis? A prospective multicenter study. Schizophr. Res. 153, 42–47. 10.1016/j.schres.2014.01.019 24508483

[B109] Veenstra-VanderWeele J. Warren Z. (2015). Intervention in the context of development: pathways toward new treatments. Neuropsychopharmacology 40, 225–237. 10.1038/npp.2014.232 25182180 PMC4262912

[B110] Wang W. Rein B. Zhang F. Tan T. Zhong P. Qin L.. (2018). Chemogenetic activation of prefrontal cortex rescues synaptic and behavioral deficits in a mouse model of 16p11.2 deletion syndrome. J. Neurosci. 38, 5939–5948. 10.1523/JNEUROSCI.0149-18.2018 29853627 PMC6021990

[B111] Warren R. P. Burger R. A. Odell D. Torres A. R. Warren W. L. (1994). Decreased plasma concentrations of the C4B complement protein in autism. Arch. Pediatr. Adolesc. Med. 148, 180–183. 10.1001/archpedi.1994.02170020066011 8118537

[B112] Weinberger D. R. Berman K. F. Zec R. F. (1986). Physiologic dysfunction of dorsolateral prefrontal cortex in schizophrenia: I. regional cerebral blood flow evidence. Arch. Gen. Psychiatry 43, 114–124. 10.1001/archpsyc.1986.01800020020004 3947207

[B113] Welham J. Isohanni M. Jones P. McGrath J. (2009). The antecedents of schizophrenia: a review of birth cohort studies. Schizophr. Bull. 35, 603–623. 10.1093/schbul/sbn084 18658128 PMC2669575

[B114] Wiesel T. N. (1982). Postnatal development of the visual cortex and the influence of environment. Nature 299, 583–591. 10.1038/299583a0 6811951

[B115] Willsey A. J. State M. W. (2015). Autism spectrum disorders: from genes to neurobiology. Curr. Opin. Neurobiol. 30, 92–99. 10.1016/j.conb.2014.10.015 25464374 PMC4586254

[B116] Woo J. J. Pouget J. G. Zai C. C. Kennedy J. L. (2020). The complement system in schizophrenia: where are we now and what’s next? Mol. Psychiatry 25, 114–130. 10.1038/s41380-019-0479-0 31439935

[B117] Xu X. Chini M. Bitzenhofer S. H. Hanganu-Opatz I. L. (2019). Transient knock-down of prefrontal DISC1 in immune-challenged mice causes abnormal long-range coupling and cognitive dysfunction throughout development. J. Neurosci. 39, 1222–1235. 10.1523/JNEUROSCI.2170-18.2018 30617212 PMC6381232

[B118] Zhao H. Tu Z. Xu H. Yan S. Yan H. Zheng Y.. (2017). Altered neurogenesis and disrupted expression of synaptic proteins in prefrontal cortex of SHANK3-deficient non-human primate. Cell Res. 27, 1293–1297. 10.1038/cr.2017.95 28741620 PMC5630686

[B119] Zhou Y. Sharma J. Ke Q. Landman R. Yuan J. Chen H.. (2019). Atypical behaviour and connectivity in SHANK3-mutant macaques. Nature 570, 326–331. 10.1038/s41586-019-1278-0 31189958

